# Incidence and outcomes of acute kidney injury in octogenarians in Jordan

**DOI:** 10.1186/s13104-018-3397-3

**Published:** 2018-05-08

**Authors:** Ashraf O. Oweis, Sameeha A. Alshelleh

**Affiliations:** 1 Division of Nephrology, Department of Medicine, Jordan University of Science and Technology, Irbid, Jordan; 2 Division of Nephrology, Department of Medicine, University of Jordan, Amman, Jordan

**Keywords:** Acute kidney injury, Aged, 80 and over, Risk factors, Outcomes, Incidence

## Abstract

**Objective:**

Improvements in the health care system, resulted in a greater number of geriatric patients diagnosed with acute kidney injury (AKI). We evaluated the incidence and outcome of AKI in octogenarians, as studies in the Middle-East region are few; moreover, treatment approaches, in addition to medical decisions, may require special consideration for advanced age to improve the outcomes.

**Results:**

At King Abdullah II teaching and referral hospital, we recruited patients aged 80–90 years who were admitted to the medical floor between January 2010 and December 2013. Patients were followed-up for at least 1 year after discharge.850 patients were admitted during the study period. Of these, 135 were excluded from our analysis. The most common admission diagnoses were uncontrolled diabetes mellitus and acute coronary syndrome. AKI occurred in 216 patients (30.2%). Using the acute kidney injury network classification; stage 1, stage 2, and stage 3 disease were present in 59, 17.5, and 23.5% of patients, respectively. Of the 115 patients who died before discharge (16.1%), 87 (75.6%) had developed AKI. Hypertension, the use of angiotensin receptor blockers and non-steroidal anti-inflammatory drugs, heart failure, and exposure to radiologic contrast media were significant risk factors for AKI.

## Introduction

Medical improvements, including the introduction of new medications, medical procedures, and treatment guidelines, lead to a longer lifespan [1]. An aging population may require new approaches for evaluation and treatment, on the other hand identifying risks for AKI which could be specific to older people may help avoid further morbidity and mortality. Studies conducted to evaluate outcomes in geriatric patients tend to treat all cases similarly regardless of the individual’s age. This practice is not necessarily a realistic approach, as we know survival in sexagenarians is different than that in nonagenarians [2, 3].

Aging causes not only functional changes, but also structural and morphological changes at the level of the kidneys, and these may increase the risk for a decline in renal function [4, 5]. With many individuals reaching the age of 80 years or above, extended life expectancy will increase the number of hospitalized geriatric patients and those with acute kidney injury [6, 7].

AKI is common in hospitalized patients, and geriatric patients have an increased risk of AKI because of their age [4, 8], multiple comorbidities, and polypharmacy among other factors [9–12], with more chance for progression of chronic kidney disease (CKD) post-AKI [13]. AKI can increase mortality and morbidity in geriatric patients regardless of the need for dialysis.

We evaluated the incidence and outcome of AKI in octogenarians in Jordan, as studies in this age group are limited, and the impact of AKI on morbidity, mortality, hospitalization and need for renal replacement therapy (RRT) needs yet to be determined and addressed in an era where the number of octogenarians and even older people are increasing in numbers in our country.

AKIN classification was used to define AKI: (stage 1 is diagnosed with a 1.5-fold increase in creatinine or more than 26.52 μmol/l, stage 2 is diagnosed with a twofold increase in creatinine, and stage 3 is diagnosed with a threefold increase in creatinine or more than 353.6 μmol/l or patient needed dialysis).

We found that risk for AKI is increasing in octogenarians with the following risk factors: hypertension (HTN), congestive heart failure (CHF), use of angiotensin-converting enzyme (ACE) Inhibitors, use of angiotensin receptor blockers (ARBs), use of nonsteroidal anti-inflammatory drugs (NSAIDs), and exposure to contrast media.

## Main text

### Methods

#### Patient selection and data collection

We retrospectively evaluated all octogenarians admitted to the medical ward of our tertiary referral teaching hospital in northern Jordan between January 2010 and December 2013. All patients had at least one year of follow-up after discharge. The first admission was analyzed if the patient had more than one admission.

Demographic data, including age, gender, comorbidities, the cause of admission, medications, and laboratory data were extracted from patients’ electronic records. Exclusion criteria were: age (< 80 years or ≥ 90 years), advanced CKD with Glomerular Filtration Rate (GFR) less than 30 ml/min in stages 4 and 5, and end stage renal disease (ESRD) patients who are already on chronic dialysis.

AKI was defined using the Acute Kidney Injury Network (AKIN) classification [14], GFR was calculated using modification of diet in renal disease (MDRD) [15], and contrast exposure was defined as intravenous contrast administration within 1 week of AKI onset. Baseline creatinine was defined as the last serum creatinine recorded before admission, as this may reflect a steadier state of kidney function.

Hypertension was defined as the use of at least one antihypertensive drug, or blood pressure > 140/90 mmHg on admission.

The Institutional Research Board of King Abdullah University Hospital approved the study. Because of the retrospective study design, the requirement for patient consent was waived.

#### Statistical analysis

All analyses were performed using STATA/MP, version 14.0 (StataCorp LLC, College Station, TX, US). Data was described using means and standard deviation (± SD) for continuous variables and percentages for categorical variables. Patients were divided into two groups: patients with and patients without AKI. The differences between the two groups in the means of continuous variables were tested using independent *t* tests. Pearson’s Chi squared test was used to compare the incidence rates of AKI according to the distribution of demographic, clinical, and relevant patient characteristics between the two groups. Cox proportional hazard model was used to assess the effect of different covariates on outcomes.

### Results

#### Patient characteristics

Of the 850 octogenarians admitted during the study period, 135 were excluded due to advanced CKD or a dialysis requirement. In the 715 remaining patients, the mean age was 85.9 years, 16.1% were diabetic, 17.5% had hypertension (HTN), and 2.8% took nonsteroidal anti-inflammatory medications (NSAIDs). Baseline characteristics are presented in Table 1.

**Table 1:** Baseline characteristics of octogenarians

Variable	N (%)
Gender	
Male	355 (49.6%)
Female	360 (50.4%)
Diabetes mellitus	115 (16.1%)
Hypertension	125 (17.5%)
Coronary artery disease	111 (15.5%)
Congestive heart failure	49 (6.9%)
Cerebral vascular accident	71 (9.9%)
Peripheral vascular disease	140 (19.9%)
Cancer	34 (4.8%)
Angiotensin converting enzyme inhibitor	191 (26.7%)
Angiotensin receptor blockers	110 (15.4%)
Non-steroidal anti-inflammatory drugs	20 (2.8%)
Contrast media exposure	153 (21.4%)
Baseline creatinine, (mean ± SD)	123.8 (105.5)

*N* number; *SD* standard deviation

The most common causes of admission were: uncontrolled diabetes mellitus (DM) with patients’ HbA1C more than 8% or random blood sugar on admission > 300 mg/dl (18.3%), acute coronary syndrome (12.8%), and other cardiac causes (6.9%) including heart failure and arrhythmias. Central nervous system disease most frequently manifested as stroke (ischemic, 5.2%; hemorrhagic, 1.9%). Infection was the cause of admission in 17.0%, most commonly urinary tract infection (6.9%) and pneumonia (2.3%). Injury secondary to a fall accounted for 5.5% of admissions.

#### Incidence and outcome of AKI

AKI was diagnosed in 216 patients (30.2%). Of these cases, 59% had stage 1 disease while stage 2 and stage 3 disease were present in 17.5% and 23.5%, respectively. One can think that the chance of having smaller changes in creatinine as in stage 1 is far more common than having bigger jumps in serum creatinine as in stages 2 and 3, which make it more sensible to find a larger number of patients in stage 1 than others.

A comparison of baseline characteristics in both groups (Table 2) revealed that patients with AKI were more likely to have HTN (*P* = 0.001) and congestive heart failure (CHF, *P* = 0.003), and to take either an angiotensin receptor blocker (ARB, *P* = 0.04) or NSAID (*P* = 0.003). Patients with AKI had been more frequently exposed to radiologic contrast media (*P* = 0.001). All the previous factors that we found are risks for AKI in the general population too, not only in octogenarians; advanced age patients may still have the tendency to have more of these risk factors together apart from age itself that has its obvious effect on kidney aging and loss of function [16, 17].

**Table 2:** Baseline characteristics of octogenarians based on acute kidney injury status

Variable	AKI (+)	AKI (−)	*P* value
Age (mean ± SD)	85.9 (1.8)	85.8 (1.8)	0.93
Diabetes mellitus	35 (16.2%)	80 (16.0%)	0.95
Hypertension	106 (21.2%)	19 (8.8%)	0.001
Coronary artery disease	34 (15.7%)	77 (15.4%)	0.92
Congestive heart failure	24 (11.1%)	25 (5.0%)	0.003
Cerebral vascular accident	21 (9.7%)	50 (10.0%)	0.90
Peripheral vascular disease	38 (17.6%)	102 (20.4%)	0.38
Cancer	9 (4.2%)	25 (5.0%)	0.62
Angiotensin converting enzyme inhibitor	66 (30.6%)	125 (25.1%)	0.12
Angiotensin receptor blockers	42 (19.4%)	68 (13.6%)	0.04
Non-steroidal anti-inflammatory drugs	12 (5.6%)	8 (1.6%)	0.003
Contrast media exposure	71 (32.9%)	82 (16.4%)	0.001
Baseline creatinine (mean ± SD)	133.9 (95.8)	119.4 (109.3)	0.10

*AKI* acute kidney injury; *SD* standard deviation

A Cox proportional hazard model demonstrated that hypertension increased the risk of AKI by 1.6 times, while NSAID use and contrast exposure increased this risk by 1.3 and 1.4 times, respectively (Table 3). The length of in-hospital stay was longer in the AKI group than in the non-AKI group: 8.9 days (± 11.8) vs. 3.5 days (± 3.6), respectively (*P* = 0.0001).

**Table 3:** Cox proportional hazard ratio

Variable	Hazard ratio	95% confidence interval	*P* value
HTN	1.6	1.07–2.4	0.02
NSAID	1.3	0.8–2.2	0.25
Contrast media exposure	1.4	1.1–1.8	0.01

HTN, hypertension; NSAID, nonsteroidal anti-inflammatory drug

By the time of discharge, the mean serum creatinine was 198.5 μmol/l (± 145) in patients with AKI and 106.4 μmol/l (± 94.6) in those without (*P* = 0.0001). Of the 115 patients who died before discharge (16.1%), 87 (75.6%) had developed AKI. In patients with AKI, mortality was significantly associated with HTN (*P* = 0.001), CHF (*P *= 0.03), and exposure to radiologic contrast media (*P* = 0.005). In summary: age, HTN, NSAID use, ARB or ACE inhibitor use, and exposure to contrast media will increase the chance to get AKI, with longer hospital stays and higher mortality especially in patients with HTN, CHF and contrast media exposure.

### Discussion

Mortality in geriatric patients is significantly associated with cardiovascular disease, and this association is even stronger in the presence of AKI [18, 19]. The incidence of AKI differs according to the definition adopted {The kidney disease improving global outcomes (KDIGO), (AKIN), risk, injury, failure, loss of kidney function, and end-stage kidney disease (RIFLE)} and according to the CKD stage in geriatric patients [14, 20]. Our sub-stratification analysis of the incidence of each stage of AKI revealed that patients with stage 1 disease (with > 0.3 mg/dl increase in creatinine) were the majority. This finding is consistent with the finding of a large population-based Chinese study that also used the AKIN criteria [21].

We found it interesting that only approximately 16 and 17% of patients had DM or HTN, respectively. We hypothesize that this is related to high cardiovascular mortality in patients with DM, which preclude the majority with this diagnosis from reaching their 80’s [22, 23], especially given the comorbidities and frailty that come with age [24, 25]. One issue to consider is the increased risk of injury due to falls in this age group. This risk is multifactorial and may occur in the presence of subtle cognitive deterioration [26], muscle weakness [27], and osteoporosis [28]. The injury would be the beginning of a sequence of complications, deterioration of function, and may require more hospitalization.

We found that risk factors for AKI include exposure to nephrotoxic agents like: NSAIDS, ACE inhibitors, ARBs, [29] and contrast media [30]. Patients with AKI had higher overall mortality (75.6% for AKI group vs. 24.4% for non-AKI group) and longer hospital stays (8.9 vs. 3.5 days) in patients without AKI. It is worth mentioning that the use of ACE inhibitors and ARB were used not only to treat HTN but also to treat those patients with coronary artery disease and CHF. Previous studies have shown that rehospitalization is also more common in octogenarians and is associated with high medical costs and mortality [24, 31]. Most of our patients, as compared to patients in other studies, had CHF or HTN [32] or were exposed to contrast media [30].

In severe AKI, the need for renal replacement therapy, including dialysis, is higher and may increase in-hospital stay, mortality, and the chance of having CKD. A significant number of affected patients may require chronic dialysis [33]. In our study, 85 patients with AKI (39.5%) needed dialysis, mainly because of hyperkalemia and pulmonary edema. Forty-two patients on dialysis (49.4%) died before discharge. The mean serum creatinine at discharge in the dialysis group was 379.1 μmol/l (± 172.6) vs. 98.9 μmol/l (± 46.9) in those that did not receive dialysis. Unfortunately, we could not follow most of the discharged dialysis patients as they were seen at different hospitals and different dialysis units.

In conclusion, prevention of AKI and early detection with reversal of cause, addressing complications and determining the need for RRT in octogenarians will improve overall survival and decrease associated morbidity in this patient population.

## Limitations

Our study was a single-center study which is the main limitation, due to study design; the cause of AKI was difficult to identify, as well as the nutritional status data, which might have a role in serum creatinine level interpretation. Although it included a fair number of patients, we may need to cooperate with our colleagues in other areas of Jordan to have more representative data about the outcomes of octogenarians nationally and facilitate the improvement of our health care system.

## Abbreviations

AKI: acute kidney injury
AKIN: acute kidney injury network
CKD: chronic kidney disease
RRT: renal replacement therapy
HTN: hypertension
DM: diabetes mellitus
CHF: congestive heart failure
ACE: angiotensin converting enzyme
ARB: angiotensin receptor blocker
NSAIDs: nonsteroidal anti-inflammatory drugs
GFR: glomerular filtration rate
ESRD: end stage renal disease
MDRD: modification of diet in renal disease
KDIGO: kidney disease improving global outcomes
RIFLE: risk injury failure loss of kidney function and end stage kidney disease
